# Two new species of the genus *Hessebius* Verhoeff, 1941 from China (Lithobiomorpha, Lithobiidae)

**DOI:** 10.3897/zookeys.735.22237

**Published:** 2018-02-06

**Authors:** Penghai Qiao, Wen Qin, Huiqin Ma, Jianping Su, Tongzuo Zhang

**Affiliations:** 1 Key Laboratory of Adaptation and Evolution of Plateau Biota, Northwest Institute of Plateau Biology, Chinese Academy of Sciences, Xining 810008. No.23 Xinning Road, Chengxi District, Xining, Qinghai, China; 2 Key Laboratory of Animal Ecological Genomics, Xining, Qinghai, China; 3 Graduate University of the Chinese Academy of Sciences, Beijing 100049, China; 4 Scientiﬁc Research Oﬃce, Hengshui University, Hengshui 053000, China

**Keywords:** COI, China, *Hessebius*, Lithobiidae, Taxonomy, the Qinghai-Tibet Plateau

## Abstract

Two new species, *Hessebius luquensis* **sp. n**. and *Hessebius ruoergaiensis* **sp. n**., are described based on material from Qinghai-Tibet Plateau. A key to the Chinese species of *Hessebius* is presented. The partial mitochondrial cytochrome c oxidase subunit I (COI) barcoding gene was amplified and sequenced for nine individuals of both species and the dataset was used for molecular phylogenetic analysis and genetic distance determination.

## Introduction

*Hessebius* was created by Verhoeff (1941) to receive two Turkish species and was substantiated by Eason (1981). Its main character is the massive expansion and projection of the dorsolateral ridge of the female gonopod, according to Zalesskaja (1978) and Eason (1981).

Presently, 13 species are known (Pei et al. 2010; Volkova 2016), mainly in Palearctic region including Central Asian (Kirghizistan, Tagikistan, Turkmenistan, Kazakistan), southern Russia, Middle East (Iran, Iraq, Armenia, Syria, Palestine), westward up to Anotalia (Toros, including the Greek southern Sporades), Cyprus, and north-east Africa (Egypt, Libya), some of which, especially those from Central Asia, were recorded only from few localities (Zapparoli 2003).

**Figure 1.**
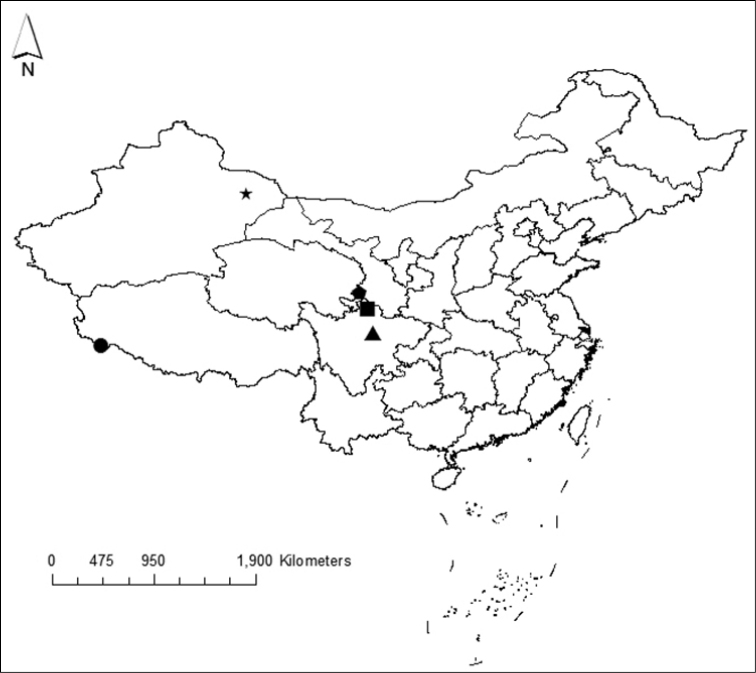
Distribution of *Hessebius* Verhoeff, 1941 in China. Symbols denote localities and species. Star = Xinjiang Uygur H *longispinipes* Ma, Pei and Zhu, 2009; circle = Tibet H *multiforaminis* Pei, Ma, Zapparoli and Zhu, 2010; triangle = Sichuan H *jangtseanus* (Verhoeff, 1942); pentagon = Luqu H *luquensis* sp. n.; square = Sichuan *H ruoergaiensis* sp. n.

The centipedes of China have been poorly studied. Up to now, three species of *Hessebius* have been recorded (Ma et al. 2014): *H. jangtseanus* (Verhoeff, 1942) distributed in Sanshenggou, Wolong Town, Wenchuan Country, Aba City, Sichuan Province; *H. longispinipes* Ma, Pei & Zhu, 2009 recorded in Xinjiang Uyghur Autonomous Region (Barkor country, Hami City) and *H. multiforaminis* Pei, Ma, Zapparoli & Zhu, 2010 recorded in Tibet Autonomous Region (Pulan country, Pulan Town, Ali City). Considering the geographic distribution of the species of *Hessebius* in China, their main habitat preference seems to be steppes, deserts or sub-deserts, and they are all seem to be native species. The known localities of *Hessebius* in China are shown in Figure 1.

## Materials and methods

*Specimen collection and preparation*: The specimens were all collected by hand, preserved in 95 % alcohol, and deposited in the collections of Northwest Institute of Plateau Biology, Chinese Academy of Sciences. Characters were examined using SZ61 Olympus stereoscope and took pictures with a SX-3 (Shanghai optical instrument factory) camera. Terminology for external anatomy follows Bonato et al. (2010). Each specimens are numbered from 1 to 12 according to collection quantity and prefix with the abbreviation of the locality. The abbreviations used are:

**T, TT** tergite, tergites;

**S, SS** sternite, sternites;

**C** coxa;

**Tr** trochanter;

**P** prefemur;

**F** femur;

**Ti** tibia;

**a** anterior;

**m** median;

**p** posterior;

**D** dorsal;

**V** ventral;

**To** Tömösváry’s organ;

**LQ** Luqu;

**REG** Ruoergai.

*DNA extraction and sequencing protocols*: Standard DNA extraction and amplification methods were performed. Total DNA was extracted from a single leg removed from each specimen samples using MicroElute Genomic DNA kit (OMEGA), after overnight incubation at 65 °C. Polymerase chain reactions (PCRs) were conducted using Mastercycler pros PCR (Eppendorff) in total reaction volumes of 39-μL volumes containing 5–60 ng template DNA, 1μL; ddH2O 28μL; 10×Buffer 5μL (Takara, Dalian, China); 0.5mm/L dNTPs 2.5μL (Takara, Dalian, China); 5U/μL Taq polymerase 0.5μL (Takara, Dalian, China); Forward Primer 1μL; Reverse Primer 1μL (synthesized by Sangon Biotech from Shanghai). An 686 bp fragment of COI was amplified using the primers LCO 1490/LCO 2198 (Edgecombe et al., 2002). PCR was performed as follows: initial denaturing at 95 °C for 10 min; followed by 35 cycles of 95 °C for 30 s, 44 °C for 30 s, and 72 °C for 90 s and a final extension at 72 °C for 10 min. The PCR products were purified using a purification kit (DC28106 250 Preps, QIAGEN, GERMAN). Sequencing reactions were implemented using ABI Prism BigdyeTM Terminator Cycle Sequencing Ready Reaction Kit on ABI 3730XL sequencer, with the PCR primers.

The GenBank accession numbers of all nine new sequences were MG515155-MG515163 (*Hessebius* COI). Sequence identities were confirmed with BLAST searches (Altschul et al. 1997). In order to eliminate indicators of nuclear mitochondrial pseudogenes (numts), such as indels, stop codons, and double peaks in sequence chromatograms, the whole dataset was translated into amino acids using the ‘invertebrate’ code in MEGA6 (Tamura et al. 2013) ; internal stop codons were absent in our dataset; gaps were absent.

*Phylogenetic analyses*: The sequences were aligned with Clustal X2.0 (Chenna et al. 2003). The aligned sequences were edited using the program BioEdit 7.0.9.0 (Hall 1999) by hand. The substitution model selection was implemented in jModelTest 2.1.4 (Darriba et al. 2012), the TIM2+G model was selected for all datasets by likelihood ratio tests either under the Akaike Information Criterion (AIC 14337.6710) or under the Bayesian Information Criterion (BIC 14617.1521). Topology was reconstructed under the TIM2+G model of nucleotide evolution in MrBayes. Bayesian inference (BI) was used to generate a phylogenetic hypothesis of the DNA haplotypes. BI was performed in MrBayes 3.2 (Ronquist and Huelsenbeck 2003) with 3,000,000 generations, sampling trees every 300 generations. Two independent runs each with four simultaneous Monte Carlo Markov chains (MCMC) were carried out. The first 25 % of generations were discarded as ‘burn-in’. The convergence of chains was confirmed until average standard deviation of split frequency is below 0.01 (0.008300) and the potential scale reduction factor (PSRF) is close to 1.0 for all parameters. In phylogenetic analysis *Anopsobius neozelanicus* Silvestri, 1909 was used as outgroup.

*Distance analysis*: The analysis involved 27 nucleotide sequences (Appendix 1). Codon positions included were 1st+2nd+3rd. All ambiguous positions were removed for each sequence pair. There were a total of 632 positions in the final dataset. Evolutionary analyses were conducted in MEGA6 (Tamura et al. 2013). All pair-wise intra- and inter-specific distances were produced to evaluate species divergence in *Hessebius*.

## Taxonomic accounts

### Class Chilopoda Latreille, 1817

#### Order Lithobiomorpha Pocock, 1895

##### Family Lithobiidae Newport, 1844

###### Subfamily Lithobiinae Newport, 1844

####### Genus *Hessebius* Verhoeff, 1941

######## Hessebius luquensis sp. n.

Taxon classificationAnimaliaLithobiomorphaLithobiidae

http://zoobank.org/9D93BD0E-90DE-4516-8C8C-BFF5BA0530CB

######### Type data.

**Figure 2.**
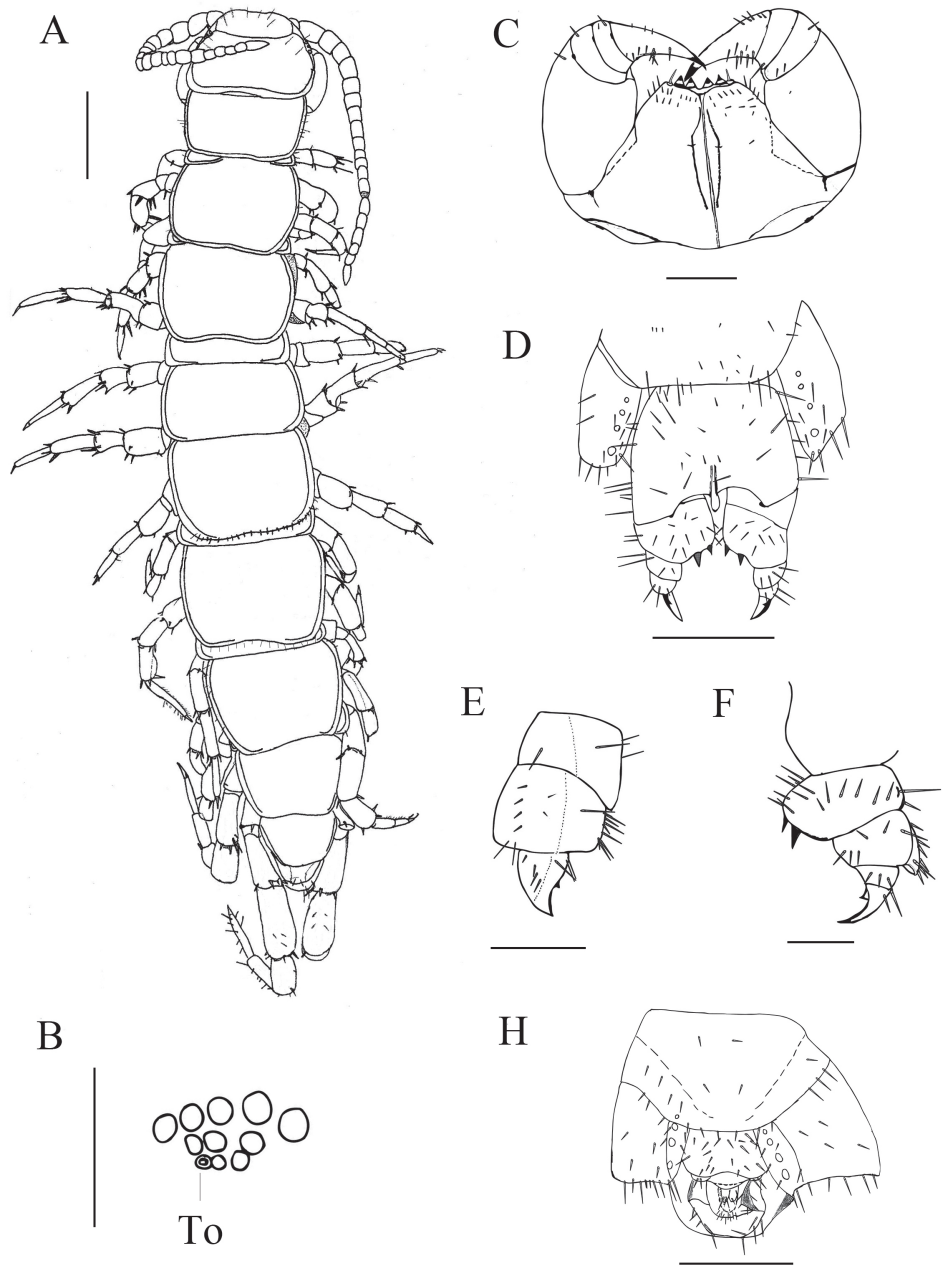
*Hessebius luquensis* sp. n., holotype, female: **A** dorsal view, scale 1 mm **B** left ocelli and Tömösváry’s organ (To), scale 250 μm; **C**, forcipular coxosternite, ventral view, scale bar 1 mm **D** posterior segments and gonopods, internal view, scale bar 500 μm **E** right gonopod, dorsal view, scale bar 250 μm **F**, right gonopod, ventral lateral view, scale bar 250 μm. Paratype, LQ 9, male **G** posterior segments and gonopods, internal view, scale bar 500 μm.

Holotype: female numbered LQ 8 (Fig. 2A–F), body length 10.4 mm, from Luqu County, the Gannan Prefecture, Gansu province, China, 34.75647°N, 102.57245°E, 13 May 2012, 3192 meters above sea level, leg. Gonghua Lin, Weiping Li. Paratypes: 8 females, 2 males, same data as holotype.

######### Habitat.

Speciemens were collected under stones along roadside on steppes from Luqu.

######### Etymology.

The name is derived from the locality Luqu where the species is discovered. Luqu country is situated in the eastern edge of the Tibetan Plateau standing on the junction of Gansu, Qinghai and Sichuan Provinces.

######### Diagnosis.

Body length 8.5–12.3 mm; head slightly widened; antennae of 20 antennomeres; 7–10 ocelli arranged in three rows; Tömösváry’s organ oval, almost equal in size to neighboring ocelli (Fig. 2B); lateral margins of forcipular coxosternite slanting; anterior margin with 2 + 2 sharp teeth and with setiform porodonts; tergites without triangular posterolateral process, a line of setae along posterior border of TT 8 and 10; legs 14 and 15 thicker than anterior ones in both sexes; a dorsal furrow on the tibia of legs 14–15 on male; coxal pores 3–6, round, arranged in one row; female gonopods with two moderately long, bullet-shaped spurs, the second article of the female gonopods having a massive process; terminal claw of the third article simple, with a small triangular protuberance on basal ventral side; male gonopods short and small.

######### Description.

Holotype (female), *body* 10.4 mm long, cephalic plate 1.3 mm width, 1.2 mm length.

*Colour* (based on specimens in 95 % ethanol): antennomeres yellow; tergites pale yellow, with brown margin; cephalic plate and T 1 brown; pleural region and sternites pale yellow; distal part of forcipules dark brown, maxillipede coxosternum and S 15 yellow; legs pale yellow with gray hue, pretarsal claw dark brown.

*Cephalic plate* smooth, convex, slightly longer than wide; short to long setae scattered along the marginal ridge of the cephalic plate; setae on head shield symmetrically arranged, three pairs between antennocellar and transverse suture, two pairs behind these sutures; frontal marginal ridge with clear transverse suture; projection of lateral marginal conspicuously discontinuous; posterior marginal ridge slightly concave without median thickening.

Ocelli (Fig. 2B): translucent with dark pigment, 1 + 4, 3, 2 ocelli on each cephalic plateau, arranged in three rows. The posterior ocellus is the biggest, seriate ocelli smaller. Tömösváry’s organ oval, nearly the same size as the adjacent ocelli, not remote, situated ventraly on anterolateral margin of cephalic pleurite.

Antennae length 3.46 mm, extending back to anterior margin of T 5, consisting of 20 elongate antennomeres covered with dense pale setae. The basal two articles enlarged, then elongate and tapering. The first article wider than long, the second article has the equal width to length, terminal article approximately 2.5 times length to width. Setation: the first two articles has fewer setae than succeeding articles especially on anterior side, then increasing, till 6 or 7, the density become constant.

Forcipular coxosternites subtrapezoidal, coxosternite with narrow, straight or slightly recurved dental margin; 2+2 teeth on dental margin, small, blunt knobs with independent sclerotization from coxosternite; porodont setiform towards its apex, much stouter than a seta at its base; no shoulders lateral to the porodont; 3 to 4 long setae along the slope, some setae scattered on ventral side of coxosternite.

Tergites smooth, T1 narrower than head and T3, subrectangular; on TT 8 and 10 there is a line of setae along posterior borders; posterior margin of TT. 1, 3, 5, 8, 10, 12, 14 a little concave, of TT. 6, 7 straight; posterior angles of all tergites rounded without triangular projections; marginal ridge narrow, entire on TT 1, 3 and 5, interrupted posteriorly on TT 7, 8, 10, 12, and 14 (Fig. 2A); tiny setae inserted in pores scattered very sparsely over the surface, more setae on anterior and posterior angles of each tergite.

Sternites trapeziform, setae scattered very sparsely on the surface; four pairs of long bristles approximately symmetrical on the anterior corner and margin, one pair on the posterior corner; among long bristles there’s small pairs of short bristles; short to long setae along posterior border, in some individuals SS 13, 14, 15 more dense.

Legs: tarsal articulations only visible with shallow ventral suture on 1^st^ to 11^th^, distinct on 12^th^ and 13^th^, well-defined on legs 14 and 15; leg pairs 14 and 15 thicker and longer with sparse setae in contrast to legs 1–13; pretarsus of legs 1–13 with a slightly curved, long, principal claw and smaller and thinner anterior and posterior accessory spines, anterior accessory spines long and slender, 0.33–0.5 the length of principal claw, posterior one stouter, 0.25 the length of principal claw, forming slightly larger angles with tarsal claws; accessory apical spines on the 14th vestigial, absent on the 15th; abundant glandular pores on surfaces of femur, tibia and tarsus of legs 14 and 15; short to moderately long setae scattered over the surface of legs 1–13, latter half of tarsi generally more setose with two rows of setae along ventral side, fewer setae on legs 14–15.

14^th^ and 15^th^ legs: swollen, 15th leg 30% of body-length, tarsus 1 4.3 times longer than wide, tarsus 2 48% length of tarsus on leg 15. Data on the leg plectrotaxy are compiled in Table 1. In the male the 14th and 15th tibia has a dorsal sulcus extending along its whole length.

**Table 1.** *Hessebius luquensis* sp. n. leg plectrotaxy; letters in brackets indicate variable spines.

Legs	Ventral					Dorsal				
	C	Tr	P	F	Ti	C	Tr	P	F	Ti
1			mp	amp	am			a(p)	a(p)	ap
2–5			mp	amp	am			ap	ap	ap
6–9			(a)mp	amp	am			ap	ap	ap
10			(a)mp	amp	am			amp	ap	ap
11			amp	amp	am			amp	ap	ap
12–13			amp	amp	am	a		amp	(a)p	ap
14		m	amp	amp	a	a		amp	(a)p	(a)p
15		m	amp	am	(a)	a		amp	p	(p)

Coxal pores on legs 12–15, circular; inner pores smaller. Distance between pores 2–3 times bigger than diameter of pore; formula 4, 4, 4, 4. Coxal pores set in a shallow groove arranged in a row with short to long setae scattered over the surface of apophysis.

Female S15 generally trapeziform, straight posteromedially; sternite of genital segment well sclerotised, wider than long; sternite of genital segment with posterior margin moderately concave between condyles of gonopods, except for a small, median approximately circular bulge, distal lightly sclerotised; short to long setae scattered over the surface of genital segment and lateral margins.

Female gonopods divided into three articles, the first article moderately broad, bearing 11–17 short to moderately long setae, arranged in three rows; the first article also bearing 2+2 moderately long, bullet-shaped spurs, inner spur slightly smaller and more anterior than the outer (Fig. 2D); the second article with 6 setae arranged in one rows(Fig. 2F); dorsolateral ridge of second article with a massive expansion projecting distally over the base of the third article (Fig. 2F), six short blunt spines along the dorsolateral ridge, one on the ventral side of dorsodistal projection (Fig. 2F); three moderately long setae on third article; dorsolateral setae one on the first article, eight on the second article four of which short and blunt and four on the third (Fig. 2E); one long dorsomedial setae on each article (Fig. 2E); terminal claw simple, slender and sharp, having small triangular protuberance on ventral side (Fig. 2D).

Male S15: subsemicircular, well chitinized, long setae scattered sparsely over its surface and posterior margins. Male genitalia: first genital sternite wider than long, well chitinized; posterior margin quite deeply concave between the gonopods, no bulge medially; 24 short to medium setae scattered sparsely over its surface and at lateral margins, second genital sternite with abundance seta; gonopod of a single small article with 2 seta on its surface, apically slightly chitinized, flat (Fig. 2H).

######### Variations.

The length of the body (from anterior to posterior) range from 8.5 mm (LQ 9) to (LQ 12) 12.3 mm. Colour of body from pale yellow to yellow brown to ferruginous. Ocelli 1 + 4, 3, 2 or 1 + 4, 2, 1 or 1 + 3, 2, 1 on the cephalic plateau. Coxal pores 4444, 4443, 5466, 6466, 5555, 4555 in female; 4444 in male. 15^th^ legs of LQ 9 (♂): length of each of the three distal articles of the 15^th^ legs in comparison with their own diameter, 15^th^ tibiae: 0.76 mm/0.31 mm = 2.45x; 15^th^ tarsus 1: 0.69 mm/0.22 mm =3.14x; 15^th^ tarsus 2: 0.62–0.13 mm/0.06 mm = 4.77x.

######### Remarks.

The female of *H. luquensis* sp. n. is mostly similar to *Hessebius longispinipes* Ma, Pei and Zhu, 2009, but can be readily distinguished by the following characters: more antennomeres (20 + 20, vs. 18 + 18 in *Hessebius longispinipes*), more ocelli in three rows, a bulge exists near the base of the porodont; 14^th^ accessory spines present, apical claw of female gonopods with triangular protuberance only on the ventral side and the apex of the male gonopod flat versus hemisphere in *H. longispinipes*.

######## Hessebius ruoergaiensis sp. n.

Taxon classificationAnimaliaLithobiomorphaLithobiidae

http://zoobank.org/46B3B393-F3C3-4D09-981E-6AC8E3136E77

######### Etymology.

The name of the species is from the type locality.

######### Holotype.

♀, numbered REG 11, China, North of Sichuan province, Ruoergai County, 33.397°N, 103.201°E, 14. V. 2012, under stones on steppe, at 3588 m above sea level, leg. Gonghua Lin, Weiping Li. Paratypes: 6 ♂, 3 ♀, same data as holotype.

######### Diagnosis.

Body length 9.2–10.0 mm; antennae composed of 19–20 antennomeres; 7–10 dark ocelli on each side; Tömösváry’s organ ovate to round, larger to the adjoining ocelli; 2+2 triangular sharp prosternal teeth; porodonts long and strong, lateral to lateral tooth; posterior angles of all tergites round; legs 14 and 15 thicker than anterior ones; coxal pores 3–5, ovate to round, arranged in one row; female gonopods with 2 bullet-shaped spurs, the second article of the female gonopods extending backwards bearing 5 lateral spines; terminal claw of the third article simple, with inconspicuous triangular ventral accessory denticles; male gonopods short and small with 2 long setae.

######### Description.

Holotype (female). Body 9.2 mm length. Cephalic plate 1.0 mm length, 1.2 mm width.

Colour: body pale yellow; antennae and distal part of forcipules brown; cephalic plate, TT 1, 2 dark and median and posterior parts of TT 3–14 dark forming a line; pleural region and SS pale yellow with dark hue; legs pale yellow with dark hue excluding tarsus yellow.

Antennae: 41.6% of body-length with 20 moderately elongate articles; the basal one wider than long; the 8, 9, 10, 11 elongate; the ultimate one is three times longer than wide. Abundance setae scattered on the surfaces of from the first to the last.

**Figure 3.**
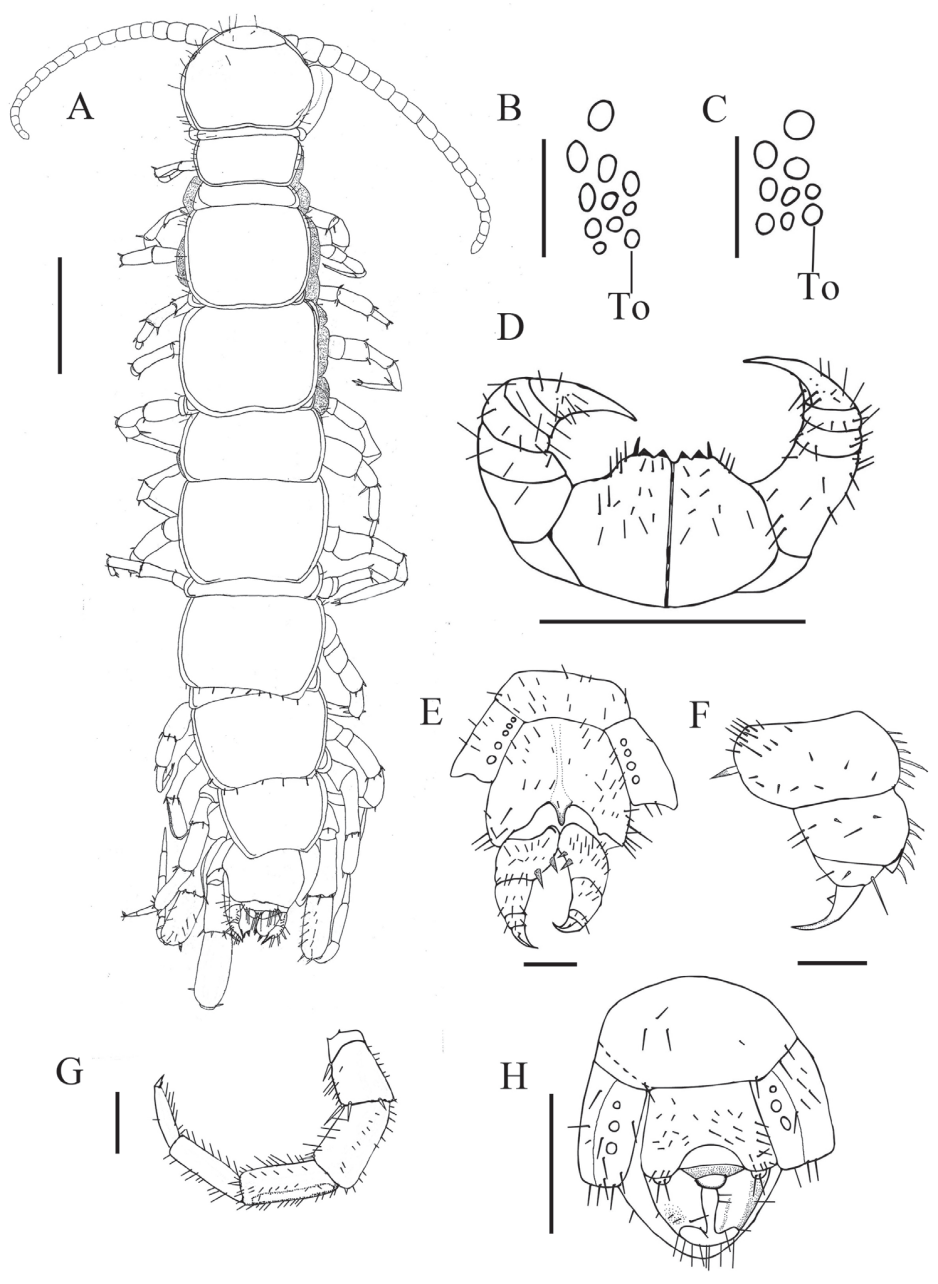
*Hessebius ruoergaiensis* sp. n., holotype, female: **A** dorsal view, scale bar 1 mm **B** left ocelli and Tömösváry’s organ (To), scale bar 250 μm **D** forcipular coxosternite, ventral view, scale bar 1 mm **E** right gonopod, dorsal view, scale bar 250 μm **C, F–H** paratype, C, REG1, female: left ocelli and Tömösváry’s organ (To), scale bar 250 μm **F** REG5, female: right gonopod, ventral lateral view, scale bar 250 μm **G** REG6, male: male left leg 15, dorsal lateral view, scale bar 500 μm **H** REG10, male: posterior segments and gonopods, internal view, scale bar 500 μm.

Cephalic plate wider than long, with clear transverse suture; median furrow on cephalic plate absent; lateral margin discontinuous, posterior margin slightly concave; moderately long setae scattered along marginal ridge and cephalic plate (Fig. 3A).

Two posterior large ocelli and eight smaller ocelli arranged in three rows (Fig. 3B). Tömösváry’s organ ovate, larger than the adjoining ocelli, some distance from the adjoining ocelli, situated on ventral side of cephalic pleurite.

Prosternum: subtrapezoidal coxosternite with narrow, straight dental margin; 2+2 subtriangular teeth as extensions of the coxosternite teeth; median incision “U” shaped; long and strong setiform porodonts; lateral borders without shoulders; pretarsal section of forcipules slightly longer than tarsal section; 3 lines of short setae and 1 moderately long setae arrange on ventral side of coxosternite (Fig. 3D).

Tergites smooth, angulation of posterolateral corners of tergites all rounded without triangular projections; T1 narrower than head and T3, concave transverse; all tergites with lateral margins; TT 1, 3 and 5 with complete posterior margins, TT 7, 8, 10, 12, and 14 incomplete; posterior margins of TT 3, 5, 8, 10, and 12 a little concave (Fig. 3A), T14 gently concave, TT1, 7, and 9 transverse, tergite of intermediate segment weakly convex. Short to long setae sparsely dispersed along lateral borders and posterior corners, a band of setae on TT 10, 12 (Fig. 3A).

Sternites smooth, S1 subsquare, SS 2–14 trapeziform, posterolateral narrower than anterolateral. One to three pairs of setae symmetrical on anterior corners; one pair of setae on posterolateral margins; a few setae distributed along posterior margins; a band of setae on anterior margins of SS 2–7.

Legs: tarsal articulation on anterior pairs of legs fused on dorsal side of leg, distinct ventrally from 1^st^ to 13^th^, on 14^th^ and 15^th^ leg divided into basitarsus and distitarsus; pretarsus claws moderately long, curved ventrally on all legs; anterior and posterior accessory spines present from the 1^st^ to the 14th leg, only posterior accessory spines on the 15th leg; anterior accessory spines long and straight, nearly half of length of the main claw, posterior accessory claws strong and curved, nearly a third length of the main claw; Legs 14–15 (Fig. 3A) thickened. Numerous short to long setae fairly evenly distributed on all sections along legs. Legs’ plectrotaxy as in Table 2. In male, one comparatively obvious furrow on the dorsal side of the tibia of legs 14 and 15 (Fig. 3G).

**Table 2.** Plectrotaxy of *Hessebius ruoergaiensis* sp. n., the holotype and paratypes. Letters in parentheses indicate variable spines.

Legs	Ventral					Dorsal				
	C	Tr	P	F	Ti	C	Tr	P	F	Ti
1			(m)p	am	am			ap	a(p)	a(p)
2–9			mp	amp	am			ap	ap	ap
10			mp	amp	am			(a)mp	ap	ap
11–12			(a)mp	amp	am	(a)		amp	ap	ap
13			(a)mp	amp	am	(a)		amp	(a)p	ap
14		m	amp	amp	am	a		amp	(a)p	(p)
15		m	amp	am	a(m)	a		amp	p	

15^th^ legs: approx. one third of body-length. Leg 15 basitarsus 129% length of distitarsus; basitarsus 84% length of tibia; tibia 2.6 times longer than maximal width, basitarsus 3.6 times, distitarsus 3.2 times. Basitarsus nearly the same length of distitarsus on leg 14.

Glandular pores: on the ventral side of femur tibia and tarsus of 14^th^ and 15^th^ legs only.

Coxal pores: on legs 12–15; set in shallow groove; the inner one smaller, circular, separated from one another by their own diameter or less; 5,5,5,5/5,5,5,4 (holotype) or 4444 in females; 4443, 3444 or 3333 in males.

Female: S15 subtrapeziform with short to long setae covered. The first genital sternite bears approx. 48 setae, posterior margin of which moderately embayed between gonopod articulations. Two long conical spurs on the female gonopod, the proximal ones smaller (Fig. 3E); Claw of female gonopod with small triangular ventral accessory denticles (Fig. 3F); five stronger and curved spines like thorn on distinct dorsodistal projection (Fig. 3F); 15 or 16 setae arranged in three rows on basal article of gonopod, six long setae on second article, 3 long setae on third (Fig. 3E).

Comparatively long setae distribute on male first genital sternite with fewer setae near S 15; posterior median margin of the first genital sternite deeply concave between gonopods; male gonopod short with two setae sometimes retracted from tergite of first genital sternite (Fig. 3H).

######### Variations.

body 9.2–10.0 mm long, cephalic plate 0.9–1.2 mm wide, 0.9–1.2 mm long; 1+3, 2, 1—1+4, 3, 2 ocelli (Fig. 3B, C); Leg 15: basitarsus 129–138% length of distitarsus, basitarsus 84–94% length of tibia; tibia 2.6–2.8 times longer than maximal width, basitarsus 3.6–3.9 times, distitarsus 3–3.2 times.

######### Remarks.

*Hessebius ruoergaiensis* sp. n. is very similar to *Hessebius jangseanus*: the number of ocelli of both species are overlapped, but *H. ruoergaiensis* has fewer ocelli, no more than ten; fewer coxal pores in *H. ruoergaiensis*, no more than five; the distribution of accessory claw on the legs is the same in both species; however, the tibia of the 14^th^ and 15^th^ leg of *H. ruoergaiensis* have dorsal sulcus which is absent in *H. jangseanus*; the plectrotaxy of legs also similar but different.

####### Key to species of the genus *Hessebius* in China

**Table:** 

1	Antennomeres 17 + 17 – 19 + 19, commonly 18 + 18	***H. longispinipes***
–	Antennomeres 20 + 20	**2**
2	Posterior accessory spinies present on the I5th leg	**3**
–	Posterior accessory spinies absent on the I5th leg	**4**
3	Dorsal sulci on 14th and I5th leg absent	***H. jangtseanus***
–	Dorsal sulci on 14th and I5th leg present	***H. ruoergaiensis***
4	Apical claw of female gonopods simple and broad	***H. multiforaminis***
–	Apical claw of female gonopods sharp and long with small triangular protuberance on ventral side	***H. luquensis***

### Molecular analysis

**Figure 4.**
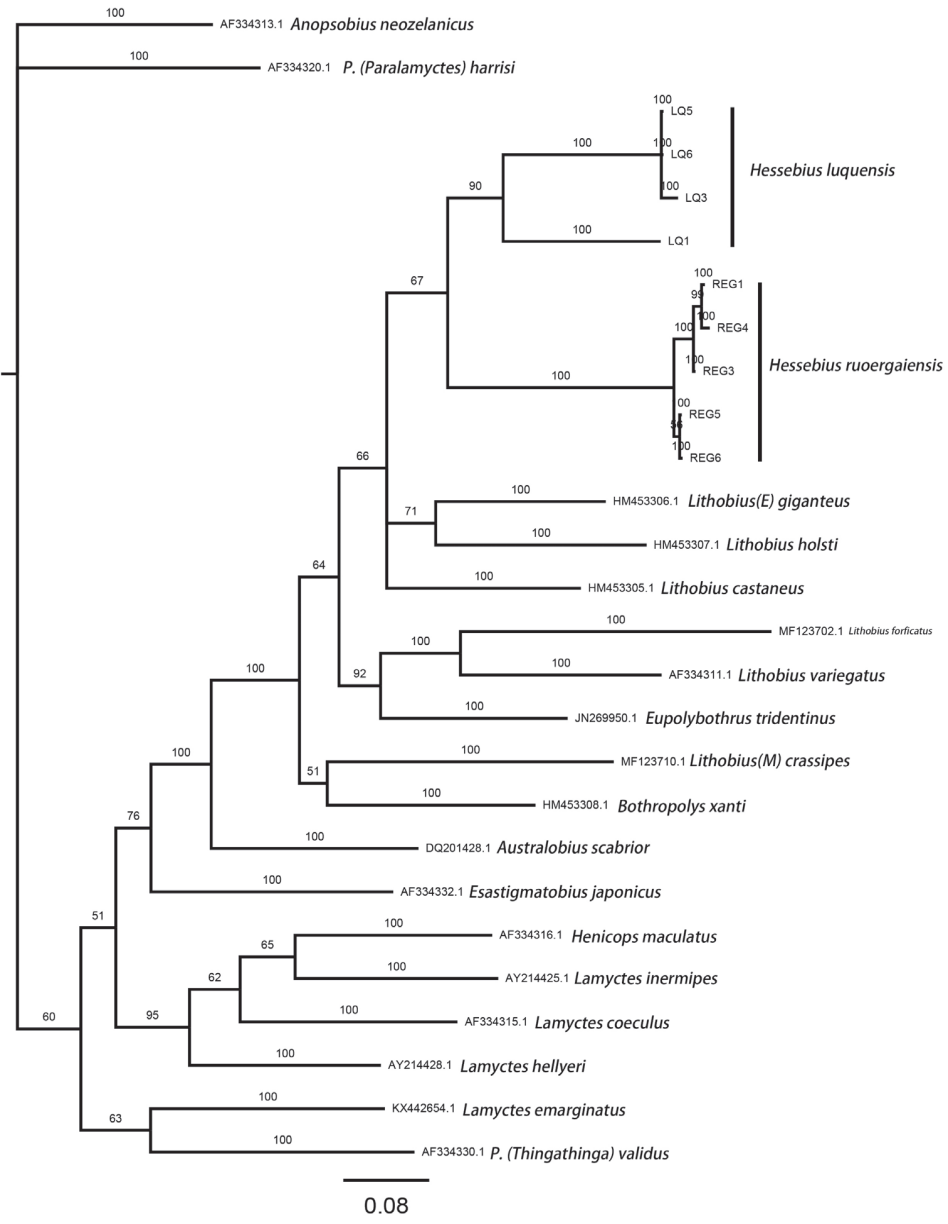
Bayesian tree for the 27 sequences of Lithobiomorpha based on COI sequences. The Bayesian posterior probabilities from Bayesian analyses are presented above the main branches. The scale bar represents substitutions per site.

The monophyly of both *Hessebius luquensis* sp. n. and *Hessebius ruoergaiensis* sp. n. is well supported with bootstrap values of 90 and 100 respectively (Fig. 4). A sister clade of *Hessebius luquensis* sp. n. and *Hessebius ruoergaiensis* sp. n. is also supported (67) (Fig. 4).

The number of base differences per site from between sequences are shown in Appendix 2. Intraspecific uncorrected p-distances range up to 6.65 % within *Hessebius luquensis* sp. n. and 0.2 % in *Hessebius ruoergaiensis* sp. n. Interspecific mean p-distance between *Hessebius luquensis* sp. n. and *Hessebius ruoergaiensis* sp. n. range is 17.3 %. Lowest interspecific distances excluding between the two new species are between *Hessebius luquensis* sp. n. and Lithobius (Ezembius) giganteus Sseliwanoff, 1881 (15.7 %) and highest between *Lithobius variegatus rubriceps* Newport, 1845 and *Lamyctes inermipes* Silvestri, 1897 (25.6 %). Uncorrected p-distances to the outgroup ranges from 17.6 % to 22.6 % (Appendix 2).

## Discussion

Both molecular analysis (Fig. 4) and morphology support that *Hessebius luquensis* sp. n. and *H. ruoergaiensis* sp. n. form new species and that the relationship is genetically close. The two species are morphologically similar, but can be readily distinguished using COI.

Generally speaking, in Lithobiomorpha, intraspecific distances are less than 10 %, while distances between species ranges often more than 10%. Sometimes the distance between the species from the same genus are larger than from different genus, such as 17.3 % (interspecific mean p-distance between *Hessebius luquensis* sp. n. and *Hessebius ruoergaiensis* sp. n.) vs 15.7 % (interspecific distances between *Hessebius luquensis* sp. n. and Lithobius (Ezembius) giganteus Sseliwanoff). This may indicate that each species have been evolved independently its habitat for a long time. Morphologically similar species, for instance species from the same genus, also have high similarity in gene that the branches which they represented joined together shown in phylogenetic tree (St. Clair and Visick 2010).

## Supplementary Material

XML Treatment for Hessebius luquensis

XML Treatment for Hessebius ruoergaiensis

## Appendix 1

**Table:** Species used for CO1 sequence analysis, sequence references, GenBank accession numbers, vouchers, and localities. ZSM = Zoologische Staatssammlung München, Germany; AM KS = vouchers in Australian Museum prefix; MCZ = Australian Museum, Sydney, Australia; SMNG = Senckenberg Museum of Natural History, Frankfurt, Germany.

Morph species name	Sequence reference	GenBank accession No.	Voucher No.	Locality
Lithobiidae				
Lithobiinae				
Lithobius (Monotarsobius) crassipes	(Voigtländer et al. 2017)	MF123710.1	SMNG VNR 17281-1	France,
Lithobius (L.) forficatus	(Voigtländer et al. 2017)	MF123702	SMNG VNR 17150-2	Germany
*Lithobius variegatus rubriceps*	(Murienne et al. 2010)	AF334311	DNA100283	Spain
Lithobius (L.) castaneus	(Murienne et al. 2010)	HM453305	DNA103939	
Lithobius (Ezembius) giganteus	(Murienne et al. 2010)	HM453306	DNA101089	
*Lithobius holsti*	(Murienne et al. 2010)	HM453307	DNA102106	
*Australobius scabrior*	(Giribet and Edgecombe 2006)	DQ201428		
Ethopolyinae				
*Eupolybothrus tridentinus*	(Stoev et al. 2013)	JN269950.1	BC ZSM MYR 00430	Croatia
*Bothropolys xanti*	(Murienne et al. 2010)	HM453308	Bmultide	
Henicopidae				
Anopsobiinae				
*Anopsobius neozelanicus*	(Edgecombe et al. 2002)	AF334313.1	AM KS 57958	New Zealand
Henicopinae				
Henicopini				
*Henicops maculatus*	(Edgecombe and Giribet 2003)	AF334316.1	AM KS57962	Australia
*Lamyctes coeculus*	(Edgecombe and Giribet 2003)	AF334315.1	DNA100288	Australia
*Lamyctes emarginatus*	(Voigtländer et al. 2017)	KX442654.1	ZSM-JSP120527-016	Germany
*Lamyctes inermipes*	(Edgecombe and Giribet 2003)	AY214425.1	DNA100478	Argentinia
*Lamyctes hellyeri*	(Edgecombe and Giribet 2003)	AY214428.1	DNA100639	Australia
Paralamyctes (P.) harrisi	(Edgecombe et al. 2002)	AF334320	AM KS 57971	New Zealand
P. (Thingathinga) validus	(Edgecombe et al. 2002)	AF334330	AM KS 57969	New Zealand
Zygethobiini				
*Esastigmatobius japonicus*	(Edgecombe et al. 2002)	AF334332	MCZ 28612	Japan

## Appendix 2

### Estimates of evolutionary divergence between sequences

**Table:** Disclaimer: Although utmost care has been taken to ensure the correctness of the caption, the caption text is provided “as is” without any warranty of any kind. Authors advise the user to carefully check the caption prior to its use for any purpose and report any errors or problems to the authors immediately (www.megasoftware.net). In no event shall the authors and their employers be liable for any damages, including but not limited to special, consequential, or other damages. Authors specifically disclaim all other warranties expressed or implied, including but not limited to the determination of suitability of this caption text for a specific purpose, use, or application.

	LQ5	LQ6	LQ3	LQ1	REG1	REG3	REG5	REG6	MF123710.1	MF123702.1	AF334311.1	JN269950.1	HM453306.1	HM453307.1	HM453305.1	HM453308.1	AF334313.1	AF334320.1	AF334316.1	AY214425.1	AF334315.1	AY214428.1	KX442654.1	AF334330.1	AF334332.1	DQ201428.1
LQ5																										
LQ6	0																									
LQ3	0	0																								
LQ1	0.133	0.133	0.133																							
REG1	0.172	0.172	0.172	0.177																						
REG3	0.172	0.172	0.172	0.177	0																					
REG4	0.172	0.172	0.172	0.177	0	0																				
REG5	0.171	0.171	0.171	0.176	0.002	0.002	0.002																			
REG6	0.171	0.171	0.171	0.176	0.002	0.002	0.002	0																		
MF123710.1	0.184	0.184	0.184	0.174	0.203	0.203	0.203	0.201	0.201																	
MF123702.1	0.191	0.191	0.191	0.209	0.215	0.215	0.215	0.214	0.214	0.222																
AF334311.1	0.18	0.18	0.18	0.199	0.204	0.204	0.204	0.203	0.203	0.225	0.207															
JN269950.1	0.172	0.172	0.172	0.176	0.191	0.191	0.191	0.19	0.19	0.195	0.199	0.177														
HM453306.1	0.157	0.157	0.157	0.165	0.168	0.168	0.168	0.166	0.166	0.201	0.206	0.182	0.16													
HM453307.1	0.19	0.19	0.19	0.171	0.191	0.191	0.191	0.19	0.19	0.203	0.225	0.19	0.187	0.172												
HM453305.1	0.16	0.16	0.16	0.153	0.182	0.182	0.182	0.18	0.18	0.188	0.204	0.199	0.168	0.18	0.169											
HM453308.1	0.174	0.174	0.174	0.187	0.214	0.214	0.214	0.212	0.212	0.204	0.214	0.201	0.18	0.196	0.203	0.19										
AF334313.1	0.21	0.21	0.21	0.206	0.228	0.228	0.228	0.226	0.226	0.21	0.214	0.222	0.176	0.218	0.187	0.185	0.21									
AF334320.1	0.217	0.217	0.217	0.228	0.229	0.229	0.229	0.228	0.228	0.253	0.223	0.229	0.225	0.223	0.226	0.21	0.226	0.179								
AF334316.1	0.225	0.225	0.225	0.234	0.239	0.239	0.239	0.239	0.239	0.231	0.239	0.253	0.22	0.226	0.245	0.206	0.233	0.191	0.196							
AY214425.1	0.241	0.241	0.241	0.231	0.237	0.237	0.237	0.239	0.239	0.239	0.261	0.256	0.223	0.22	0.229	0.222	0.234	0.203	0.217	0.16						
AF334315.1	0.215	0.215	0.215	0.218	0.218	0.218	0.218	0.218	0.218	0.209	0.229	0.239	0.212	0.207	0.218	0.206	0.226	0.179	0.212	0.168	0.176					
AY214428.1	0.223	0.223	0.223	0.228	0.248	0.248	0.248	0.247	0.247	0.222	0.237	0.234	0.206	0.231	0.215	0.212	0.223	0.161	0.193	0.168	0.172	0.169				
KX442654.1	0.215	0.215	0.215	0.212	0.228	0.228	0.228	0.226	0.226	0.21	0.233	0.241	0.217	0.233	0.236	0.217	0.233	0.187	0.206	0.18	0.188	0.184	0.182			
AF334330.1	0.229	0.229	0.229	0.247	0.247	0.247	0.247	0.245	0.245	0.223	0.223	0.244	0.217	0.25	0.236	0.234	0.258	0.19	0.196	0.199	0.215	0.19	0.188	0.185		
AF334332.1	0.225	0.225	0.225	0.244	0.245	0.245	0.245	0.244	0.244	0.231	0.242	0.234	0.191	0.218	0.231	0.233	0.206	0.184	0.199	0.177	0.199	0.195	0.185	0.204	0.188	
DQ201428.1	0.199	0.199	0.199	0.203	0.223	0.223	0.223	0.222	0.222	0.207	0.222	0.201	0.187	0.199	0.22	0.199	0.206	0.191	0.188	0.196	0.188	0.199	0.19	0.196	0.217	0.187
